# Yellow-y Functions in Egg Melanization and Chorion Morphology of the Asian Tiger Mosquito, *Aedes albopictus*

**DOI:** 10.3389/fcell.2021.769788

**Published:** 2021-12-16

**Authors:** Mi Young Noh, Seulgi Mun, Karl J. Kramer, Subbaratnam Muthukrishnan, Yasuyuki Arakane

**Affiliations:** ^1^ Department of Forest Resources, AgriBio Institute of Climate Change Management, Chonnam National University, Gwangju, South Korea; ^2^ Department of Applied Biology, Chonnam National University, Gwangju, South Korea; ^3^ Department of Biochemistry and Molecular Biophysics, Kansas State University, Manhattan, KS, United States

**Keywords:** tyrosine metabolism, *yellow* (dopachrome conversion enzyme), melanization, pigmentation, egg, chorion, RNA interference (RNAi), *Aedes albopictus*

## Abstract

The Asian tiger mosquito, *Aedes albopictus*, is one of the most serious public health pests, which can transmit various vector-borne diseases. Eggs from this mosquito species become dark black shortly after oviposition and exhibit high desiccation resistance. Some of the Yellow proteins that act as dopachrome conversion enzymes (DCEs) are involved in the tyrosine-mediated tanning (pigmentation and sclerotization) metabolic pathway that significantly accelerates melanization reactions in insects. In this research, we analyzed the function of one of the *yellow* genes, *yellow-y* (*AalY-y*), in eggshell/chorion melanization of *Ae. albopictus* eggs. Developmental and tissue-specific expression measured by real-time PCR showed that *AalY-y* transcripts were detected at all stages of development analyzed, with significantly higher levels in the ovaries from blood-fed adult females. Injection of double-stranded RNA for *AalY-y* (ds*AalY-y*) had no significant effect on fecundity. However, unlike ds*EGFP-*treated control eggs that become black by 2–3 h after oviposition (HAO), ds*AalY-y* eggs were yellow-brown at 2 HAO, and reddish-brown even at 48 HAO. ds*EGFP* eggs exhibited resistance to desiccation at 48 HAO, whereas approximately 50% of the ds*AalY-y* eggs collapsed when they were moved to a low humidity condition. In addition, TEM analysis revealed an abnormal morphology and ultrastructure of the outer-endochorion in the ds*AalY-y* eggs. These results support the hypothesis that AalY-y is involved in the tyrosine-induced melanin biosynthetic pathway, plays an important role in black melanization of the chorion and functions in conferring proper morphology of the outer-endochorion, a structure that is presumably required for egg desiccation resistance in *Ae. albopictus*.

## Introduction

The Asian tiger mosquito, *Aedes albopictus*, one of the most serious vectors of human diseases, is known to transmit dengue virus, Zika virus, yellow fever virus and chikungunya virus (Weaver and Reisen, 2010; Sukhralia et al., 2019; Lwande et al., 2020). Previous studies have suggested that eggshell melanization and sclerotization (tanning) and/or serosal cuticle formation in several mosquito species are important for egg resistance to desiccation, including *Aedes* species (Li and Christensen, 1993; Li, 1994; Li et al., 1996; Kim et al., 2005; Li and Li, 2006; Goltsev et al., 2009; Wu et al., 2013; Farnesi et al., 2017). Eggs from *Aedes* mosquitos, including *Ae. albopictus*, are pale and soft right at the time of oviposition. However, shortly thereafter, they become darker (pigmented/melanized) and harder (sclerotized) over a period of several hours, and exhibit high desiccation resistance, which is a major factor that helps them to survive and aid in their rapid spread throughout the world (Benoit et al., 2010; Faull et al., 2016; Diniz et al., 2017; Schmidt et al., 2018).

**FIGURE 1:**
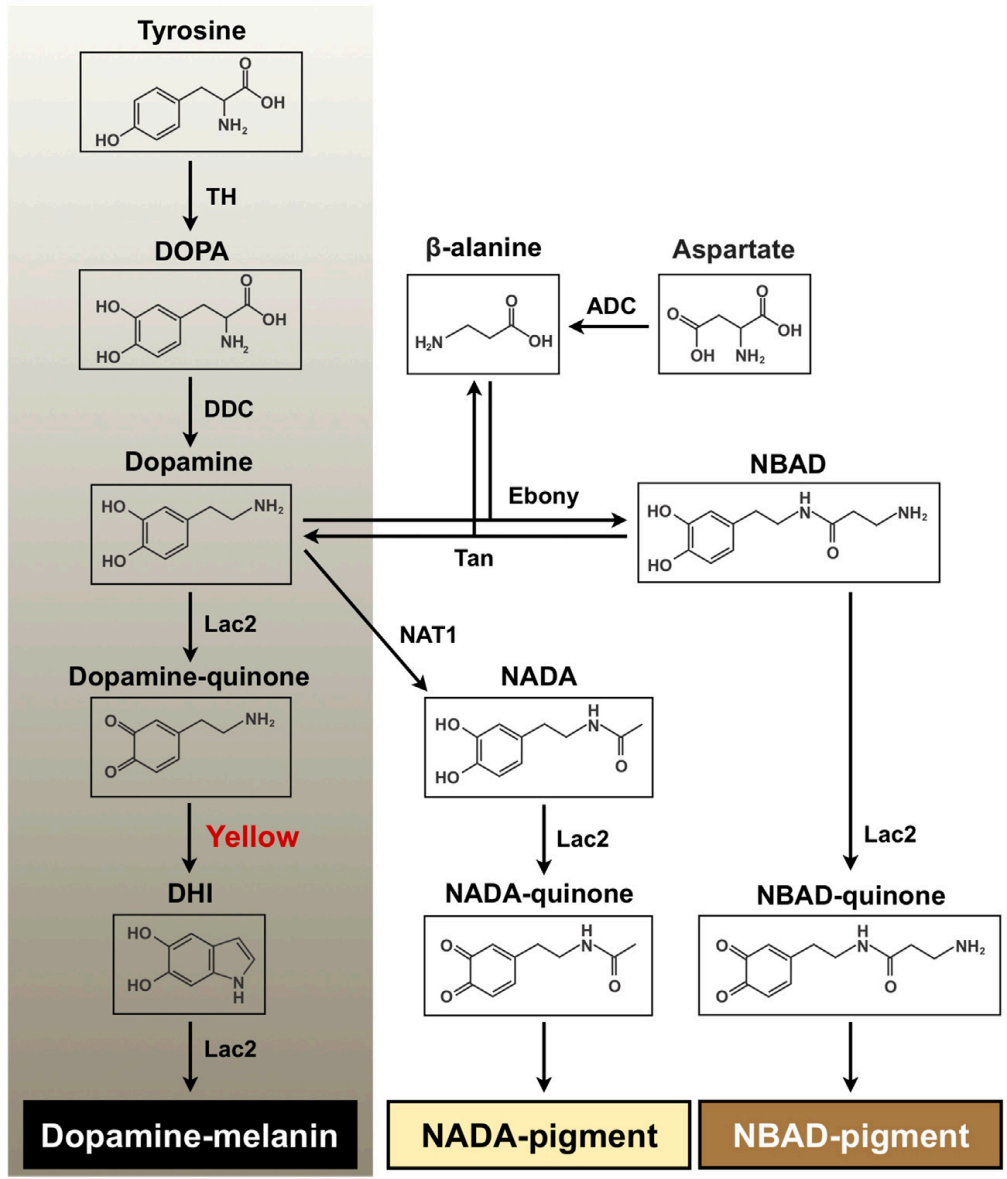
Proposed melanin and quinoid pigment biosynthetic pathway. Tyrosine and dopamine are major precursors for synthesis of black melanin and *N*-acylquinoid-derived pigments. Yellow protein, which converts dopaminechrome to dihydroxyindole (DHI) and accelerates melanin biosynthesis, is highlighted in red. DOPA, 3,4-dihydroxyphenylalanine; Dopamine, 4-(2-aminoethyl)benzene-1,2-diol; DHI, 5, 6-dihydroxyindole; NBAD, *N*-β-alanyldopamine; NADA, *N*-acetyldopamine; TH, tyrosine hydroxylase; DDC, DOPA decarboxylase; Lac2, laccase 2; ADC, aspartate 1-decarboxylase; Ebony, *N*-β-alanyldopamine synthase; Tan, NBAD hydrolase; NAT1, arylalkylamine *N*-acetyltransferase 1.

In insects, tanning is a complex and vital physiological process in coloration and hardening of cuticle and eggshell as well as in wound healing and encapsulation of entomopathogens during an immune response (Sugumaran, 2002; Andersen, 2010). With tyrosine as the initial substrate, melanin biosynthesis includes the hydroxylation of tyrosine to 3,4-dihydroxyphenylalanine (DOPA) by tyrosine hydroxylase (TH) and the decarboxylation of DOPA to dopamine by DOPA decarboxylase (DDC) (Figure 1). Dopamine is oxidized to dopamine-quinone by a phenoloxidase, Laccase 2 (Lac2), which is further converted to dihydroxyindole (DHI) by the dopachrome conversion enzyme (DCE, Yellow). DHI is oxidized by Lac2 to DHI-chrome (melanochrome), which is then polymerized to form melanic pigment (Andersen, 2010; Arakane et al., 2016; Noh et al., 2016b; Futahashi and Osanai-Futahashi, 2021). Furthermore, dopamine undergoes an *N*-acylation reaction to form *N*-acetyldopamine (NADA) or *N*-β-alanylation to *N*-β-alanyldopamine (NBAD). This is followed by oxidation of NADA and NBAD to NADA-quinone and NBAD-quinone, respectively, which are polymerized to their corresponding *N*-acylquinoid-derived pigments (Figure 1). These *N*-acylquinones, in addition, are also utilized as cross-linking agents between structural cuticular proteins for cuticle sclerotization (Kramer et al., 1984; Kramer et al., 2001; Andersen, 2010; Mun et al., 2015; Noh et al., 2016a).

*Yello*w comprises a rapidly evolving gene family generating functionally diverse paralogs in insects, and its products have been classified into at least ten subgroups (Ferguson et al., 2011). One of the critical functions of *yellow* family genes is insect body coloration. Some *yellow* genes appear to encode DCEs that are involved in melanin biosynthesis in the tyrosine-mediated tanning pathway (see Figure 1). For instance, Han et al. (2002) demonstrated that recombinant Yellow-f (DmY-f) and Yellow-f2 (DmY-f2) protein from *Drosophila melanogaster* (fruit fly) exhibited a DCE activity, which accelerates melanin synthesis. In particular, a functional importance of *yellow-y* (*Y-y*) in cuticle pigmentation/melanization appears to be highly conserved among insects. A reduction in function of *Y-y* caused lighter and yellowish cuticle, including species-specific body markings/spots in many insect species from several orders, such as Diptera, Lepidoptera, Coleoptera, Hymenoptera, Hemiptera, and Blattodea (Wittkopp et al., 2002; Futahashi et al., 2008; Arakane et al., 2010; Shirataki et al., 2010; Riedel et al., 2011; Liu et al., 2016; Heinze et al., 2017; Chen et al., 2018; Paulo et al., 2019; Liu et al., 2020; Mun et al., 2020; Wang et al., 2020; Han et al., 2021; Nguyen et al., 2021; Nie et al., 2021; Shirai et al., 2021).

Recent studies using the CRISPR/Cas9 system suggested that *Y-y* possesses diverse functions besides melanin production for body coloration. For example, a *BtY-y* knockout mutant of *Bactrocera tryoni* (Queensland fruit fly) showed a significantly decreased adult eclosion rate and a lower percentage of fliers from the resulting adults (Nguyen et al., 2021). Unlike that observed in the *DmY-y* mutant of *D. melanogaster* (Drapeau et al., 2003; Massey et al., 2019), there was no significant difference in courtship and copulation between the *BtY-y* mutant and wild-type strain of *B. tryoni*. For lepidopteran species, *BaY-y* is required for normal morphology of wing scales of the tropical butterfly *Bicyclus anynana* (Matsuoka and Monteiro, 2018). In *Spodoptera frugiperda* (fall armyworm), *SfY-y* knockout caused a significant reduction in copulation, oviposition and egg hatching (Han et al., 2021). In *Spodoptera litura* (tobacco cutworm), similarly, *SlY-y* knockout mutant embryos failed to hatch, but they grew and developed normally once they were removed manually from the eggshell (Shirai et al., 2021). In contrast, embryos from an *AiY-y* knockout mutant of *Agrotis ipsilon* (black cutworm) did hatch out; however, a dehydration-like phenotype was observed in the resulting larvae (Chen et al., 2018). The *Y-y* mutants of all lepidopteran species described above exhibited light yellowish larval and adult body color, but there was no obvious difference in pigmentation of the pupal cuticle between the *Y-y* mutants and their counterpart wild-type strains. In contrast, knockout of *PxY-y* in *Plutella xylostella* (diamondback moth) caused yellowish larval, pupal and adult body coloration but did not affect oviposition and egg hatching (Wang et al., 2020).

Although *Y-y* genes involved in the tyrosine-derived tanning pathway are critical for body coloration in many insect species, their physiological function on pigmentation/melanization and integrity of the eggshell have not been well elucidated. In this study, we report the characterization and investigation of the functional importance of *AalY-y* on melanization and morphology of *Ae. albopictus* eggs. Using dsRNA-mediated gene silencing (RNAi) and transmission electron microscopy (TEM) analysis, we demonstrate that *AalY-y* is required for eggshell darkening and normal morphology of the outer-endochorion, both of which appear to be critical for egg hatching, embryo viability and egg resistance to desiccation in *Ae. albopictus*.

## Materials and Methods

### Mosquito Rearing

*Ae. albopictus* was reared at 27 ± 0.5 °C and 80% relative humidity (rh) under a photoperiod of 14 h light: 10 h dark. Larvae and adults were fed with ground fish food (TetraMin Baby, Melle) and 10% sucrose, respectively. For colony maintenance and RNAi experiments, adult females were artificially blood-fed with sheep blood (Carolina Biological Supply Company) by using a glass mosquito feeder (Chemglass).

### Cloning of *AalY-y* cDNA

The *Ae. albopictus* ortholog of *D. melanogaster* (DmY-y; accession number: NP_476792) was identified by performing a BLAST search of the *Ae. albopictus* genome. To clone a *AalY-y* cDNA, total RNA was isolated from females 48 h post-blood-feeding (n = 10) by using the RNeasy Mini Kit (Qiagen). First-strand cDNAs were synthesized with the SuperScript III First-strand Synthesis System (Invitrogen), using an oligo-(dT) primer according to the manufacturer’s instructions. Primers 5′-ATG TGG AAG TCG GTG GTC TG-3′ and 5′-CTA GTA CAG CTG CTT CCA G-3′ that included the predicted start and stop codons, respectively, were used to amplify the coding sequence for *AalY-y* (1,662 bp) by PCR. The cDNA fragment was cloned into the pGEM-T vector (Promega) and sequenced. The GenBank accession number of the *AalY-y* clone is MN702767.

### Protein Sequence Analysis

Yellow-y-like proteins in insect genomes were identified via a BLAST search in the NCBI database, using the AalY-y protein sequence as query. Protein sequences were analyzed for signal peptides, using the SignalP 5.0 server (http://www.cbs.dtu.dk/services/SignalP). Major Royal Jelly Protein (MRJP) domains were identified, using the Conserved Domain Database (CDD, http://www.ncbi.nlm.nih.gov). *N*-glycosylation sites were predicted, using the NetNGlyc 1.0 server (http://www.cbs.dtu.dk/services/NetNGlyc/). Multiple sequence alignment of deduced amino acid sequences of the Yellow-y proteins was made, using the ClustalW software tool (http://www.ebi.ac.uk/Tools/msa/clustalw2). See Supplementary Table S1 for the accession numbers of Yellow-y proteins used for the amino acid sequence alignment.

### Real-Time PCR

To analyze expression patterns of *AalY-y* during development, total RNA was isolated from pools of whole insects (n = 10, except for embryos and larvae) at various developmental stages, including embryos, larvae, pupae, sugar-fed adult males and females, and blood-fed females (36–48 h after blood feeding), followed by cDNA synthesis as described above. To analyze induction patterns of *AalY-y* in response to the ingestion of blood, total RNA was isolated from sugar-fed females or from blood-fed females collected at 12, 24, 36, 42, 48, 60, 72, 84, and 96 h following blood or sugar feeding (n = 10 for each time point). To analyze tissue-specific expression of *AalY-y*, total RNA was isolated from ovaries and carcasses (whole body minus ovary) dissected from 48 h post-blood-fed females (n = 10). Total RNA was independently isolated for each of the three biological replicates. Real-time PCR were performed using the primers 5′-TCG AGC ACA GCT TCT TCT TC-3′ and 5′-GAC AGG GAC ATT CCG AAG ATA C-3′ in a 40 μL reaction volume containing 1 μL of template cDNA, 20 μL TB green™ Premix Ex Taq (TAKARA), 0.25 μM of each primer, using the Thermal Cycler Dice real-time PCR system (TAKARA). Real-time PCR was carried out with an initial denaturation at 95°C for 30 s, followed by 40 cycles of 95°C for 5 s and 60°C for 30 s. At the end of the PCR reaction, a melting curve was generated to evaluate the possibility of undesirable side products. The threshold cycle number (Ct) was determined and used for comparative quantitative (2^−ΔΔCt^) analyses. The primer set consisting of 5′-ACA AGC TGC GTC ACT TCT ACG ACA-3′ and 5′-CTT GTC GTT TCC ACC GGC AAT CTT-3′ was used to amplify transcripts for *Ae. albopictus* ribosomal protein S6 (*AalRpS6*) (accession number: AF154066) as the reference gene to normalize for differences between the concentrations of cDNA templates (Noh et al., 2020).

### Synthesis of Double-Stranded RNA (dsRNA)

dsRNA for *AalY-y* was synthesized according to the protocol described previously (Arakane et al., 2005). The template for synthesis of ds*AalY-y* (464 bp) was amplified by PCR using the primers 5’-(T7)-CAC ACT ACA TCT CGA GTG-3′ and 5’-(T7)-GAT GTG TAC TTG GTC ACT-3’. T7 indicates the T7 polymerase recognition sequence. ds*AalY-y* was synthesized by using the AmpliScribe™ T7-Flash™ Transcription kit (Epicentre Technologies). dsRNAs for *AalY-g*, *AalY-g2*, and for the enhanced green fluorescent protein (ds*EGFP*) were synthesized as described previously (Noh et al., 2020).

### RNA Interference (RNAi)

RNAi was carried out as described previously (Noh et al., 2020). ds*AalY-y* (approximately 1 μg per insect) were injected into the lateral thorax of 3 days-old females ∼2 h following blood-feeding (n = 15 for each of the three independent experiments). ds*EGFP* was injected to serve as a negative control. After injection, dsRNA-treated females were maintained at 27 ± 0.5°C and 70% rh and allowed to lay eggs 4 days after treatment. Eggs were collected immediately after oviposition for further experimentation. To analyze the knockdown level of *AalY-y* transcripts, cDNAs were synthesized from total RNA isolated from adults 48 h after dsRNA treatment (n = 5). Total RNA was independently isolated for each of the three biological replicates.

### Transmission Electron Microscopy (TEM)

The ultrastructure of the chorion was analyzed by TEM as described previously (Noh et al., 2020). Eggs obtained from dsRNA-treated females were collected 1, 3, 24 and 48 h after oviposition (HAO) and fixed in a mixture of 0.1% glutaraldehyde and 4% paraformaldehyde in 0.1 M sodium cacodylate buffer (pH 7.4) for 24 h at room temperature. Samples were rinsed three times for 15 min with 0.1 M sodium cacodylate buffer (pH 7.4), and then dehydrated in a progressive ethanol gradient of 50, 60, 70, 80, 90, 95, and 100% for 20 min each. The tissues were infiltrated in LR white resin (Electron Microscopy Sciences) (1:1 ethanol:resin for 4 h, 1:2 ethanol:resin overnight and 100% resin for 4 h). The tissues were vacuum-infiltrated for 2 h, embedded in gelatin capsules (Electron Microscopy Sciences), and then the resin was polymerized at 55°C for 12–14 h, followed by ultrathin sectioning. Ultrathin sections (∼90 nm) were stained with 4% aqueous uranyl acetate for 10 min and then imaged, using a transmission electron microscope (JEM-1400, JEOL).

### Egg Resistance to Desiccation Under Low Humidity Conditions

Desiccation resistance of eggs was analyzed according to the protocol described previously (Valencia et al., 1996; Rezende et al., 2008). Approximately 300–400 eggs obtained from dsRNA-treated females (n = 13–15) at 48 HAO were transferred to filter paper and then air-dried at room temperature and 40–50% rh for 30 min. The number of collapsed eggs was counted under a stereomicroscope (Leica M80).

### Data Analysis

Statistical analyses were performed using PSPP-1.4.0. A one-way ANOVA followed by the Tukey’s honestly significant difference (HSD) test was used to compare data from multiple samples. The Student *t*-test was used to analyze statistical difference between two samples (pairwise comparison).

## Results and Discussion

### *AalY-y* cDNA Sequence

The *Ae. albopictus* ortholog (AalY-y) of *D. melanogaster* Yellow-y protein (DmY-y) was identified by performing a BLAST (*tblastn* program) search of the *Ae. albopictus* genome database. Primers encoding the predicted start and stop codon regions were used to amplify a 1,662 bp cDNA that included the full-open reading frame. The predicted amino acid sequence is 593 residues with a theoretical molecular mass of 62.1 kDa and a predicted pI of 5.75. Consistent with our expectation that Yellow is a secretory protein, AalY-y contains a putative signal peptide sequence in addition to a conserved ∼285 amino acid-long MRJP domain (Supplementary Figure S1). *Y-y* appears to be a single-copy gene in all species analyzed except for *Bactrocera* species such as *B. tryoni*, *B. dorsalis* and *B. latifrons* that have two genes encoding Y-y-like proteins (denoted Y-y1 and Y-y2 by Nguyen et al. (2021)) (Supplementary Figure S2). The amino acid sequence identities and similarities of AalY-y with other insect homologs range from 51 to 95% and 76–98%, respectively (Supplementary Table S2). As shown in Supplementary Figure S3, the multiple amino acid sequence alignment of the AalY-y sequence with its orthologs from several species belonging to different orders of insects indicated that Yellow-y proteins from Diptera and Lepidoptera analyzed, including *D. melanogaster* (DmY-y), *Musca domestica* (MdY-y), *B. tryoni* (BtY-y1 and -y2), *B. dorsalis* (BdY-y1 and -y2), *B. latifrons* (BlY-y1 and -y2), *Aedes aegypti* (AaeY-y), *Anopheles gambiae* (AgaY-y), *Bombyx mori* (BmY-y), *Papilio xuthus* (PxY-y), *S. litura* (SlY-y), *S. frugiperda* (SfY-y), *P. xylostella* (PxyY-y) and *A. ipsilon* (AiY-y) have a C-terminal extension of 85–151 amino acids, whereas this extension is absent in Yellow-y orthologs from Coleoptera, Hymenoptera and Hemiptera, such as *Tribolium castaneum* (TcY-y), *Tenebrio molitor* (TmY-y), *Leptinotarsa decemlineata* (LdY-y), *Apis mellifera* (AmY-y), *Nasonia vitripennis* (NvY-y), *Oncopeltus fasciatus* (OfY-y) and *Platymeris biguttatus* (PbY-y), suggesting that Yellow-y proteins in Diptera and Lepidoptera have gained the additional C-terminal stretch rather recently in evolutionary time. All of Yellow-y proteins analyzed have one to three putative *N*-glycosylation sites in the MRJP domain and/or the C-terminal stretch (Supplementary Figure S3).

### *AalY-y* Expression

**FIGURE 2:**
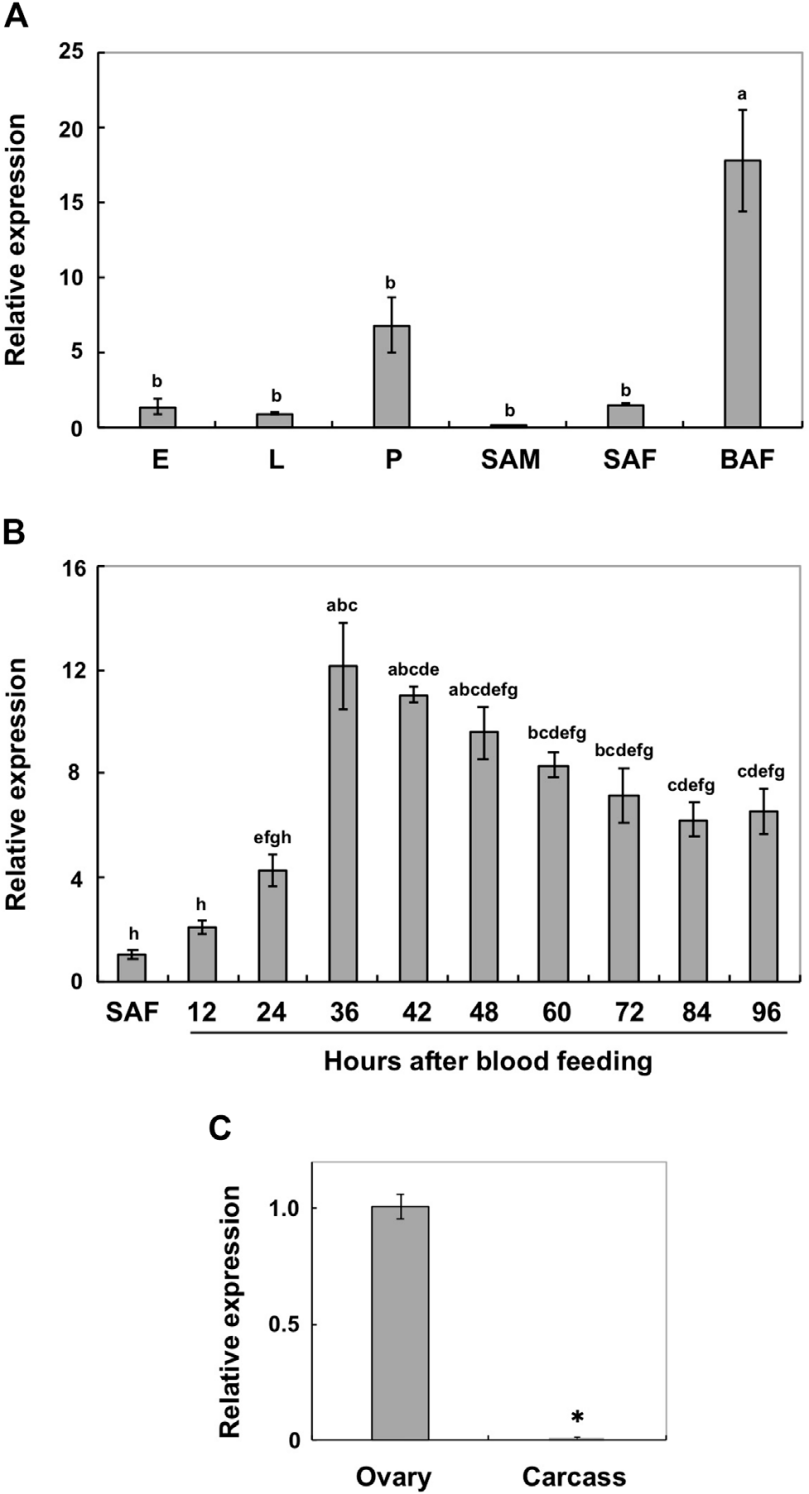
Expression profiles of *AalY-y* by real-time PCR. Transcript levels of *AalY-y* relative to that of *Ae. albopictus* ribosomal protein S6 (*AalRpS6*) were determined by real-time PCR. Data are shown as the mean values ± SE (n = 3). **(A)** Expression patterns of *AalY-y* during development. E, eggs; L, larvae; P, pupae; SAM, sugar-fed adult males; SAF, sugar-fed adult females; BAF, blood-fed adult females (36–48 h post blood meal). Expression levels for *AalY-y* are presented relative to the levels of expression in the eggs (E). Different letters above each bar indicate significant differences (*p* < 0.05, Tukey HSD test). **(B)** Induction patterns of *AalY-y* in response to a blood meal. Total RNA was isolated from SAF and BAF 12, 24, 36, 42, 48, 60, 72, 84 and 96 h after blood feeding. Expression levels for *AalY-y* are presented relative to the levels of expression in the sugar-fed adult females (SAF). Different letters above each bar indicate significant differences (*p* < 0.05, Tukey HSD test). **(C)** To analyze tissue-specific expression of *AalY-y* in adults, total RNA was extracted from ovaries of 48 h post blood-fed females. The remaining tissues (whole body minus ovary) were pooled as the carcass. Expression levels for *AalY-y* are presented relative to the levels of expression in the ovaries. An asterisk indicates a significant difference in transcript levels of *AalY-y* (*p* = 0.006, *t*-test) between samples.

Real-time PCR was performed to analyze the expression patterns of *AalY-y* during *Ae. albopictus* development. *AalY-y* transcripts were detected at all stages of development analyzed from embryo to adult stages, with trace levels detected in sugar-fed adult males, but the levels were substantially higher in blood-fed adult females (Figure 2A). Thus, we further analyzed the induction patterns of *AalY-y* in response to blood feeding. As shown in Figure 2B, the expression of *AalY-y* gradually increased, with the highest levels 36 h after blood feeding and then gradually decreased thereafter by the end of our sampling period (96 h after blood feeding). To assess the tissue-specific expression of *AalY-y* in the blood-fed females, we dissected 48 h post-blood fed-females to obtain ovary and carcass (whole body without ovary) tissues. The transcripts of *AalY-y* were detected in the ovaries but not in the carcass (Figure 2C), indicating that in adult females of *Ae. albopictus*, *AalY-y* is specifically expressed in the ovaries after blood-feeding.

*Yellow-y* transcripts have also been detected in ovaries from other mosquito species, and its protein products were present in extracts from the eggshells of *Ae. aegypti* and *An. gambiae* (Amenya et al., 2010; Marinotti et al., 2014). In addition, *Yellow-g* (*Y-g*) and *yellow-g2* (*Y-g2*), which comprise one of the *yellow* gene subfamilies, appear to be specifically expressed in ovaries and may have a role in eggshell/chorion rigidity in many insect species, such as *Ae. albopictus*, *D. melanogaster*, *Ae. aegypti*, *An. gambiae* and the German cockroach, *Blattella germanica* (Claycomb et al., 2004; Irles et al., 2009; Amenya et al., 2010; Dissanayake et al., 2010; Marinotti et al., 2014; Noh et al., 2020). Our results suggest that, like *yellow-g* and *-g2*, ovary-specific expression of *AalY-y* in blood-fed adult females might participate in eggshell formation, particularly in the dark black melanin production of *Ae. albopictus* eggs.

### Loss-of-Function Egg Phenotypes

**FIGURE 3:**
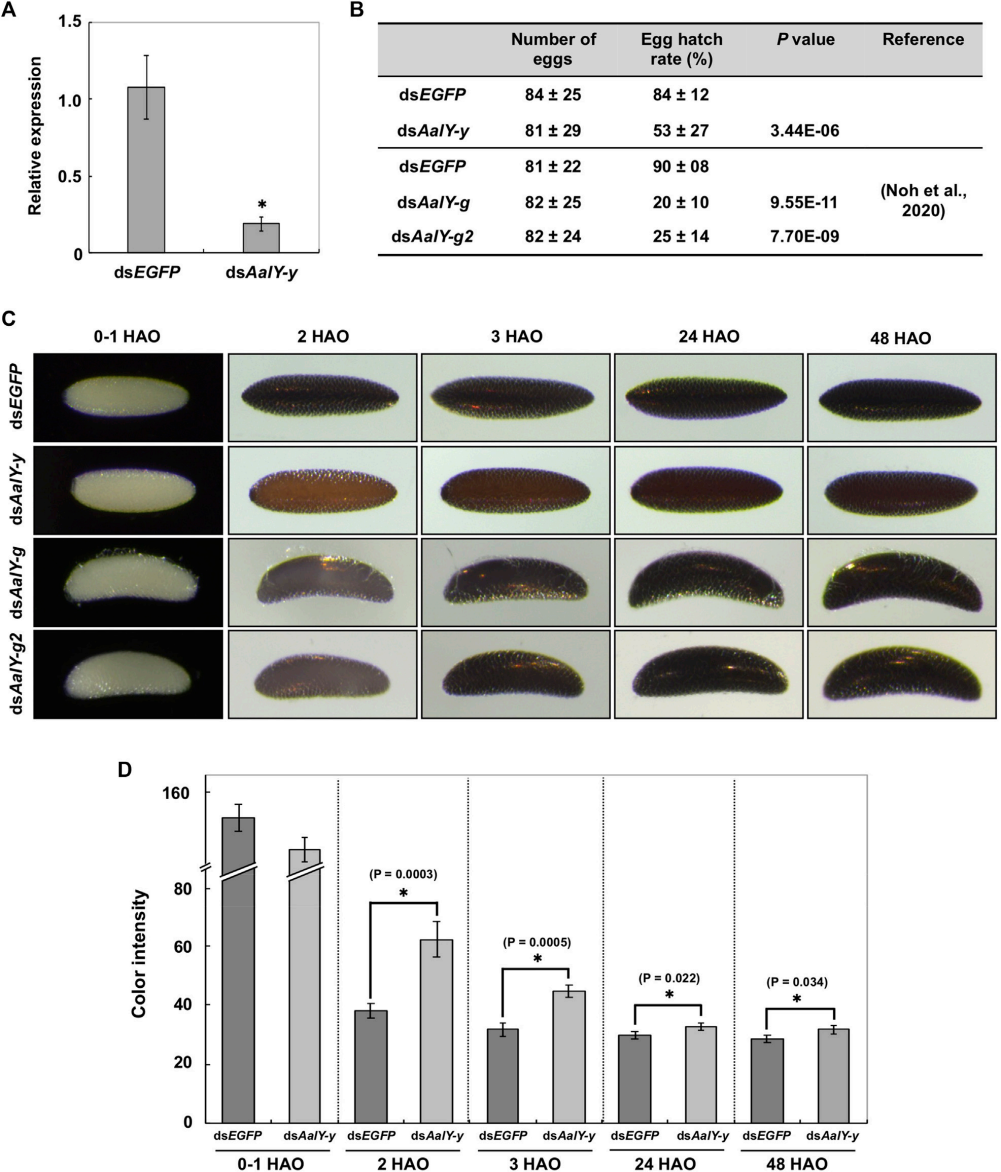
Loss-of-function egg phenotypes produced by RNAi of *AalY-y*. ds*AalY-y* or ds*EGFP* (1 μg per insect) was injected into 3 day-old adult females shortly after blood-feeding. **(A)** Total RNA was isolated from adults (n = 5) 48 h after dsRNA treatment to analyze knockdown levels of *AalY-y* transcripts. Expression levels of *AalY-y* in ds*AalY-y*-treated insects are presented relative to their levels in ds*EGFP*-treated control. An asterisk indicates a significant difference in transcript levels of *AalY-y* between control and test animals (*p* = 0.01, *t*-test). Data are shown as the mean value ± SE (n = 3). **(B)** Injection of ds*AalY-y* had no significant effect on fecundity (*p* > 0.36, *t*-test), while it decreased egg hatching rate by nearly a half (*p* < 3.44E-06, *t*-test). Data are shown as the mean value ± SE (n = 17–27). However, as seen following RNAi of *AalY-g* and *AalY-g2* (Noh et al., 2020), a significantly decreased egg hatch rate was observed in ds*AalY-y*-treated insects. **(C)** dsRNAs for *AalY-g* and *AalY-g2* | were synthesized and injected as previously reported (Noh et al., 2020). Melanization of either ds*AalY-g* or ds*AalY-g2* eggs was delayed for 2 h after oviposition (HAO) with the eggshell/chorion, eventually becoming a dark black color similar to 3 HAO ds*EGFP* control eggs. In contrast, ds*AalY-y* eggs exhibited a light yellow-brown color at 2 HAO and were still reddish brown even 48 HAO. **(D)** The color intensity in equivalent regions of the eggshell of 0–1, 2, 3, 24 and 48 HAO eggs from dsRNA-treated adult females was determined as mean gray values (average luminance) using ImageJ software as described previously (Noh et al., 2015). Under this measurement, the lower color intensity indicates the darker eggshell coloration/melanization. Data are shown as the mean values ± SE (n = 9–11). Asterisk indicates a significant difference in color intensity of the eggshell (*t*-test) between ds*EGFP* control and ds*AalY-y* eggs.

RNAi was used to determine whether AalY-y is required for eggshell melanization. Injection of dsRNA for *AalY-y* (ds*AalY-y*) into adult females shortly after blood-feeding led to a substantial decrease in transcript levels of *AalY-y* compared to that in ds*EGFP*-treated control animals (Figure 3A) without any significant effect on their fecundity (Figure 3B). However, in contrast to a hatch rate of 84% for eggs from the ds*EGFP*-treated females, the hatching rate of eggs from ds*AalY-y-*treated females decreased significantly to only 53%. We recently reported that injection of either ds*AalY-g* or ds*AalY-g2* into adult females had no effect on their fecundity, whereas the resulting eggs exhibited an obviously lower hatch rate (Noh et al., 2020). These results suggest that, like AalY-g and AalY-g2, AalY-y is important for egg hatching and/or embryo viability. Similarly, reduced egg hatch rates were also observed in *Y-y* knockout mutants of two *Spodoptera* species, *S. litura* (Shirai et al., 2021) and *S. frugiperda* (Han et al., 2021). In the former, SlY-y-deficient embryonic larvae grew and developed normally, once they were dissected out manually from eggshells, suggesting that SlY-y may play a role in rigidity of the mandibles, which is critical for chewing out of the eggshell/chorion during egg hatching in *S. litura*.

Eggs from either ds*EGFP*- or ds*AalY-y*-treated *Ae. albopictus* females were pale by 1 h after oviposition (HAO). However, the former became dark black by 2–3 HAO, while the latter had a light yellow-brown color at 2 HAO and were only reddish-brown even at 48 HAO, which was significantly lighter in color than its counterpart ds*EGFP* eggs (Figures 3C,D). As we reported previously (Noh et al., 2020), both ds*AalY-g* and ds*AalY-g2* eggs exhibited a delayed initial melanization by 2 HAO with the eggshell eventually becoming dark black by ∼3 HAO, as had been observed in ds*EGFP* control eggs (bottom two panels in Figure 3C). These results indicate that although AalY-g and AalY-g2 are required for rapid eggshell melanization soon after oviposition, AalY-y also plays a major role in melanin production for black eggs of *Ae. albopictus*. In contrast, the shape of ds*AalY-y* eggs, like that seen in the ds*EGFP* control ones, was spindle-like, whereas ds*AalY-g* and ds*AalY-g2* eggs were abnormally crescent- and blimp-shaped, with the outermost exochorion being more fragile and partially peeled off (Figure 3C). These results suggest that, unlike AalY-g and AalY-g2, AalY-y is not important for the rigidity of the eggshell or the integrity of the exochorion of the *Ae. albopictus* egg.

### AalY-y Is Required for Normal Morphology of the Outer-Endochorion

The *Ae. albopictus* eggshell, like that seen in many other mosquito species, is composed of two maternally-derived layers, exochorion and endochorion, secreted by follicle cells, as well as the innermost chitinous serosal cuticle layer that is secreted by the zygotically-derived extra-embryonic serosal cells during embryogenesis (Rezende et al., 2008; Goltsev et al., 2009; Farnesi et al., 2015; Noh et al., 2020). In *Ae. albopictus*, the endochorion consists of two morphologically distinct layers, a highly electron-dense outer-endochorion (OE) and a less electron-dense monotonous inner-endochorion (IE) (Noh et al., 2020).

**FIGURE 4:**
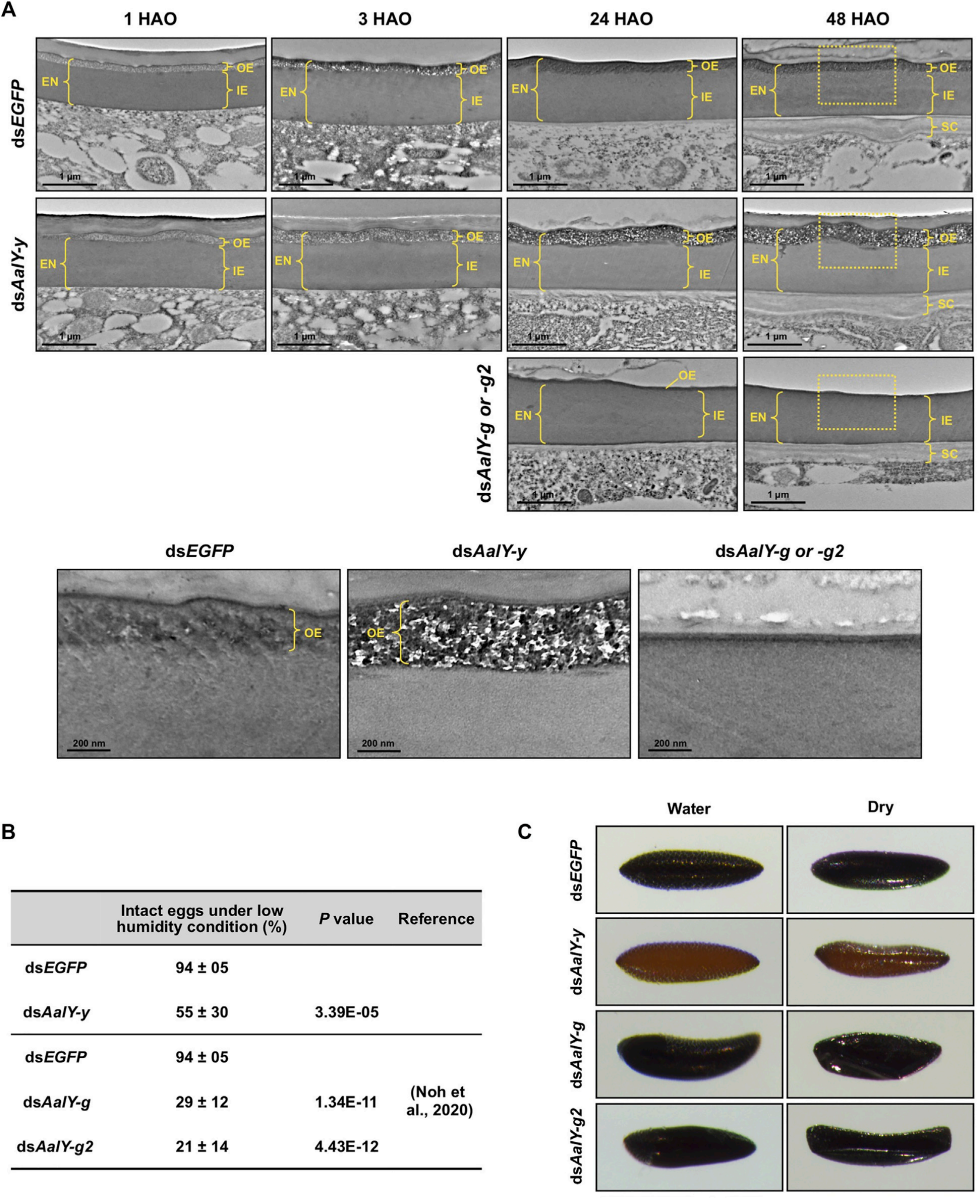
Ultrastructure of eggshell and resistance to desiccation of ds*AalY-y* eggs. **(A)** Ultra-thin sections (∼90 nm) of 1, 3, 24 and 48 h after oviposition (HAO) eggs from ds*AalY-y* or ds*EGFP*-treated females were prepared for analysis of the ultrastructure of the eggshell by TEM. The outer-endochorion (OE) layer of ds*EGFP* eggs exhibited an electron-lucent spotty morphology at one HAO, which became electron-dense with obscure morphology by 24 HAO. In contrast, the OE of ds*AalY-y* eggs remained electron-lucent at three HAO and exhibited an electron-lucent and electron-dense spotty morphology at 24 and 48 HAO. Representative images of 24 and 48 HAO eggs from either ds*AalY-g*- or ds*AalY-g2*-treated insects were also shown. Little or no electron-dense OE was evident in those eggs. The outer portions of the 48 HAO eggs (boxed) are enlarged in the bottom panels. EN, endochorion; OE, outer endochorion; IE, inner endochorion; SC, serosal cuticle. **(B)** Egg resistance to desiccation was analyzed at 48 HAO. Data are shown as the mean value ± SE (300–400 eggs obtained from 13 to 15 female adults). **(C)** The 48 HAO eggs before (Water) and after 30 min under low humidity condition (Dry) are shown.

We tested for ultrastructural changes of ds*AalY-y* eggs by TEM. In the ds*EGFP* control eggs, as seen in the wild-type strain of *Ae. albopictus*, the OE showed an electron-lucent spotty morphology at 1 HAO, which became electron-dense at 3 HAO and very electron-dense with obscure morphology at 24 HAO and beyond (Figure 4A), probably due to a progressive tanning of this layer. Similarly, the electron-lucent spotty OE was evident in 1 HAO ds*AalY-y* eggs (Figure 4A). However, unlike the result observed with the ds*EGFP* eggs, the OE was still electron-lucent at 3 HAO and exhibited an abnormally electron-lucent and electron-dense spotty morphology at 24 HAO and even at a later stage (48 h) of embryonic development (Figure 4A). There was no obvious difference in morphology between the IE layers of ds*EGFP* and ds*AalY-y* eggs.

We reported recently that *Ae. albopictus* eggs collected by 18 HAO collapsed shortly after transfer to low humidity conditions, whereas more than 90% of the 24 HAO and later eggs remained intact, indicating that the eggs acquired tolerance to desiccation by 24 HAO under our rearing conditions (Noh et al., 2020). We further analyzed the desiccation resistance of the ds*AalY-y* eggs at 48 HAO. Like that seen in eggs obtained from the wild-type strain of *Ae. albopictus*, ds*EGFP*-control eggs exhibited high desiccation resistance at 48 HAO, whereas nearly half of the ds*AalY-y* eggs collapsed when they were moved to a low-humidity condition (Figures 4B,C). In addition, the 48 HAO eggs from either ds*AalY-g*- or ds*AalY-g2*-treated females, in which little or no electron-dense OE was evident (Figure 4A), showed rather poor desiccation resistance, with approximately 70–80% of the eggs collapsing under a low-humidity condition (Figures 4B,C) (Noh et al., 2020). These results indicate that, like AalY-g and AalY-g2, AalY-y is required for normal morphology and the formation of the OE, a structure that is critical for egg resistance to desiccation in *Ae. albopictus*.

In this work, we demonstrated that AalY-y, involved in the tyrosine-induced melanin biosynthetic pathway, plays an important role in black melanization of the eggshell/chorion as well as functions in the maintenance of the proper morphology of the OE, egg hatch/embryo viability and egg resistance to desiccation in *Ae. albopictus*. Our TEM studies indicate that the key tissue/structure affected by the deficiency of AalY-y protein is the outer-endochorion layer of the egg, which presumably acts as a barrier against water loss. This is the first evidence that a Yellow-y protein functions in normal eggshell integrity and formation, which is critical for insect reproduction, in addition to cuticle pigmentation and/or morphology. Our results provide further support for the hypothesis that different members of the family of *yellow* genes affect not only melanin-type pigment production but also participate in different, but essential physiological roles in the maintenance of eggshell integrity, function and viability.

## Data Availability

The datasets presented in this study can be found in online repositories. The names of the repository/repositories and accession number(s) can be found in the article/Supplementary Material.

## References

[B1] Amenya D. A. Chou W. Li J. Yan G. Gershon P. D. James A. A. (2010). Proteomics Reveals Novel Components of the *Anopheles gambiae* Eggshell. J. Insect Physiol. 56 (10), 1414–1419. 10.1016/j.jinsphys.2010.04.013 20433845 PMC2918668

[B2] Andersen S. O. (2010). Insect Cuticular Sclerotization: a Review. Insect Biochem. Mol. Biol. 40 (3), 166–178. 10.1016/j.ibmb.2009.10.007 19932179

[B3] Arakane Y. Dittmer N. T. Tomoyasu Y. Kramer K. J. Muthukrishnan S. Beeman R. W. (2010). Identification, mRNA Expression and Functional Analysis of Several *Yellow* Family Genes in *Tribolium castaneum* . Insect Biochem. Mol. Biol. 40 (3), 259–266. 10.1016/j.ibmb.2010.01.012 20149870

[B4] Arakane Y. Muthukrishnan S. Kramer K. J. Specht C. A. Tomoyasu Y. Lorenzen M. D. (2005). The *Tribolium Chitin Synthase* Genes *TcCHS1* and *TcCHS2* Are Specialized for Synthesis of Epidermal Cuticle and Midgut Peritrophic Matrix. Insect Mol. Biol. 14 (5), 453–463. 10.1111/j.1365-2583.2005.00576.x 16164601

[B5] Arakane Y. Noh M. Y. Asano T. Kramer K. J. (2016). “Tyrosine Metabolism for Insect Cuticle Pigmentation and Sclerotization,” in Extracellular Composite Matrices in Arthropods. Editors Cohen E. Moussian B. (Cham: Springer), 165–220. 10.1007/978-3-319-40740-1_6

[B6] Benoit J. B. Lopez-Martinez G. Phillips Z. P. Patrick K. R. Denlinger D. L. (2010). Heat Shock Proteins Contribute to Mosquito Dehydration Tolerance. J. Insect Physiol. 56 (2), 151–156. 10.1016/j.jinsphys.2009.09.012 19782687 PMC2861860

[B7] Chen X. e. Cao Y. Zhan S. Zhang Y. Tan A. Huang Y. (2018). Identification of *Yellow* Gene Family in *Agrotis ipsilon* and Functional Analysis of *Aiyellow-y* by CRISPR/Cas9. Insect Biochem. Mol. Biol. 94, 1–9. 10.1016/j.ibmb.2018.01.002 29337139

[B8] Claycomb J. M. Benasutti M. Bosco G. Fenger D. D. Orr-Weaver T. L. (2004). Gene Amplification as a Developmental Strategy. Developmental Cell 6 (1), 145–155. 10.1016/S1534-5807(03)00398-8 14723854

[B9] Diniz D. F. A. de Albuquerque C. M. R. Oliva L. O. de Melo-Santos M. A. V. Ayres C. F. J. (2017). Diapause and Quiescence: Dormancy Mechanisms that Contribute to the Geographical Expansion of Mosquitoes and Their Evolutionary success. Parasites Vectors 10 (1), 310. 10.1186/s13071-017-2235-0 28651558 PMC5485599

[B10] Dissanayake S. N. Ribeiro J. M. Wang M.-H. Dunn W. A. Yan G. James A. A. (2010). aeGEPUCI: a Database of Gene Expression in the Dengue Vector Mosquito, *Aedes aegypti* . BMC Res. Notes 3, 248. 10.1186/1756-0500-3-248 20920356 PMC2958886

[B11] Drapeau M. D. Radovic A. Wittkopp P. J. Long A. D. (2003). A Gene Necessary for normal Male Courtship,yellow, Acts Downstream Offruitless in the Drosophila Melanogaster Larval Brain. J. Neurobiol. 55 (1), 53–72. 10.1002/neu.10196 12605459

[B12] Farnesi L. C. Menna-Barreto R. F. S. Martins A. J. Valle D. Rezende G. L. (2015). Physical Features and Chitin Content of Eggs from the Mosquito Vectors *Aedes aegypti, Anopheles aquasalis* and *Culex quinquefasciatus*: Connection with Distinct Levels of Resistance to Desiccation. J. Insect Physiol. 83, 43–52. 10.1016/j.jinsphys.2015.10.006 26514070

[B13] Farnesi L. C. Vargas H. C. M. Valle D. Rezende G. L. (2017). Darker Eggs of Mosquitoes Resist More to Dry Conditions: Melanin Enhances Serosal Cuticle Contribution in Egg Resistance to Desiccation in *Aedes, Anopheles* and *Culex* Vectors. Plos Negl. Trop. Dis. 11 (10), e0006063. 10.1371/journal.pntd.0006063 29084225 PMC5679640

[B14] Faull K. J. Webb C. Williams C. R. (2016). Desiccation Survival Time for Eggs of a Widespread and Invasive Australian Mosquito Species, *Aedes* (Finlaya) *Notoscriptus* (Skuse). J. Vector Ecol. 41 (1), 55–62. 10.1111/jvec.12194 27232125

[B15] Ferguson L. C. Green J. Surridge A. Jiggins C. D. (2011). Evolution of the Insect *Yellow* Gene Family. Mol. Biol. Evol. 28 (1), 257–272. 10.1093/molbev/msq192 20656794

[B16] Futahashi R. Osanai-Futahashi M. (2021). “Pigments in Insects,” in Pigments, Pigment Cells and Pigment Patterns. Editors Hashimoto H. Goda M. Futahashi R. Kelsh R. Akiyama T. (Singapore: Springer), 3–43. 10.1007/978-981-16-1490-3_1

[B17] Futahashi R. Sato J. Meng Y. Okamoto S. Daimon T. Yamamoto K. (2008). *Yellow* and *Ebony* Are the Responsible Genes for the Larval Color Mutants of the Silkworm *Bombyx mori* . Genetics 180 (4), 1995–2005. 10.1534/genetics.108.096388 18854583 PMC2600937

[B18] Goltsev Y. Rezende G. L. Vranizan K. Lanzaro G. Valle D. Levine M. (2009). Developmental and Evolutionary Basis for Drought Tolerance of the *Anopheles gambiae* Embryo. Developmental Biol. 330 (2), 462–470. 10.1016/j.ydbio.2009.02.038 PMC4084816 19298808

[B19] Han Q. Fang J. Ding H. Johnson J. K. Christensen B. M. Li J. (2002). Identification of *Drosophila melanogaster* Yellow-f and Yellow-f2 Proteins as Dopachrome-Conversion Enzymes. Biochem. J. 368 (Pt 1), 333–340. 10.1042/BJ20020272 12164780 PMC1222967

[B20] Han W. Tang F. Zhong Y. Zhang J. Liu Z. (2021). Identification of *Yellow* Gene Family and Functional Analysis of *Spodoptera frugiperda Yellow-y* by CRISPR/Cas9. Pestic. Biochem. Physiol. 178, 104937. 10.1016/j.pestbp.2021.104937 34446204

[B21] Heinze S. D. Kohlbrenner T. Ippolito D. Meccariello A. Burger A. Mosimann C. (2017). CRISPR-Cas9 Targeted Disruption of the Yellow Ortholog in the Housefly Identifies the *Brown Body* Locus. Sci. Rep. 7, 4582. 10.1038/s41598-017-04686-6 28676649 PMC5496933

[B22] Irles P. Bellés X. Piulachs M. D. (2009). Identifying Genes Related to Choriogenesis in Insect Panoistic Ovaries by Suppression Subtractive Hybridization. BMC Genomics 10, 206. 10.1186/1471-2164-10-206 19405973 PMC2683872

[B23] Kim S. R. Yao R. Han Q. Christensen B. M. Li J. (2005). Identification and Molecular Characterization of a Prophenoloxidase Involved in *Aedes aegypti* Chorion Melanization. Insect Mol. Biol. 14 (2), 185–194. 10.1111/j.1365-2583.2004.00547.x 15796752 PMC2881666

[B24] Kramer K. J. Kanost M. R. Hopkins T. L. Jiang H. Zhu Y. C. Xu R. (2001). Oxidative Conjugation of Catechols with Proteins in Insect Skeletal Systems. Tetrahedron 57 (2), 385–392. 10.1016/s0040-4020(00)00949-2

[B25] Kramer K. J. Morgan T. D. Hopkins T. L. Roseland C. R. Aso Y. Beeman R. W. (1984). Catecholamines and β-alanine in the Red Flour Beetle, *Tribolium castaneum* . Insect Biochem. 14, 293–298. 10.1016/0020-1790(84)90063-5

[B26] Li J. Christensen B. M. (1993). Involvement of L-Tyrosine and Phenol Oxidase in the Tanning of *Aedes aegypti* Eggs. Insect Biochem. Mol. Biol. 23 (6), 739–748. 10.1016/0965-1748(93)90048-W

[B27] Li J. (1994). Egg Chorion Tanning in *Aedes aegypti* Mosquito. Comp. Biochem. Physiol. A: Physiol. 109 (4), 835–843. 10.1016/0300-9629(94)90231-3 7828027

[B28] Li J. Hodgeman B. A. Christensen B. M. (1996). Involvement of Peroxidase in Chorion Hardening in *Aedes aegypti* . Insect Biochem. Mol. Biol. 26 (3), 309–317. 10.1016/0965-1748(95)00099-2 8900599

[B29] Li J. S. Li J. (2006). Major Chorion Proteins and Their Crosslinking during Chorion Hardening in *Aedes aegypti* Mosquitoes. Insect Biochem. Mol. Biol. 36 (12), 954–964. 10.1016/j.ibmb.2006.09.006 17098170 PMC1885465

[B30] Liu J. Lemonds T. R. Marden J. H. Popadić A. (2016). A Pathway Analysis of Melanin Patterning in a Hemimetabolous Insect. Genetics 203 (1), 403–413. 10.1534/genetics.115.186684 26984060 PMC4858788

[B31] Liu X. L. Han W. K. Ze L. J. Peng Y. C. Yang Y. L. Zhang J. (2020). Clustered Regularly Interspaced Short Palindromic repeats/CRISPR-Associated Protein 9 Mediated Knockout Reveals Functions of the *Yellow-y* Gene in *Spodoptera litura* . Front. Physiol. 11, 615391. 10.3389/fphys.2020.615391 33519520 PMC7839173

[B32] Lwande O. W. Obanda V. Lindström A. Ahlm C. Evander M. Näslund J. (2020). Globe-Trotting *Aedes aegypti* and *Aedes albopictus*: Risk Factors for Arbovirus Pandemics. Vector-Borne Zoonotic Dis. 20 (2), 71–81. 10.1089/vbz.2019.2486 31556813 PMC7041325

[B33] Marinotti O. Ngo T. Kojin B. B. Chou S.-P. Nguyen B. Juhn J. (2014). Integrated Proteomic and Transcriptomic Analysis of the *Aedes aegypti* Eggshell. BMC Developmental Biol. 14, 15. 10.1186/1471-213X-14-15 PMC4234484 24707823

[B34] Massey J. H. Chung D. Siwanowicz I. Stern D. L. Wittkopp P. J. (2019). The *Yellow* Gene Influences *Drosophila* Male Mating success through Sex Comb Melanization. eLife 8, e49388. 10.7554/eLife.49388 31612860 PMC6794089

[B35] Matsuoka Y. Monteiro A. (2018). Melanin Pathway Genes Regulate Color and Morphology of Butterfly wing Scales. Cell Rep. 24 (1), 56–65. 10.1016/j.celrep.2018.05.092 29972791

[B36] Mun S. Noh M. Y. Kramer K. J. Muthukrishnan S. Arakane Y. (2020). Gene Functions in Adult Cuticle Pigmentation of the Yellow Mealworm, *Tenebrio molitor* . Insect Biochem. Mol. Biol. 117, 103291. 10.1016/j.ibmb.2019.103291 31812474

[B37] Mun S. Young Noh M. Dittmer N. T. Muthukrishnan S. Kramer K. J. Kanost M. R. (2015). Cuticular Protein with a Low Complexity Sequence Becomes Cross-Linked during Insect Cuticle Sclerotization and Is Required for the Adult Molt. Sci. Rep. 5, 10484. 10.1038/srep10484 25994234 PMC4440208

[B38] Nguyen T. N. M. Mendez V. Ward C. Crisp P. Papanicolaou A. Choo A. (2021). Disruption of Duplicated *Yellow* Genes in *Bactrocera tryoni* Modifies Pigmentation Colouration and Impacts Behaviour. J. Pest Sci. 94, 917–932. 10.1007/s10340-020-01304-9

[B39] Nie H. Y. Liang L. Q. Li Q. F. Li Z. H. Zhu Y. N. Guo Y. K. (2021). CRISPR/Cas9 Mediated Knockout of *Amyellow-y* Gene Results in Melanization Defect of the Cuticle in Adult *Apis mellifera* . J. Insect Physiol. 132, 104264. 10.1016/j.jinsphys.2021.104264 34081960

[B40] Noh M. Y. Kim S. H. Gorman M. J. Kramer K. J. Muthukrishnan S. Arakane Y. (2020). Yellow-g and Yellow-g2 Proteins Are Required for Egg Desiccation Resistance and Temporal Pigmentation in the Asian Tiger Mosquito, *Aedes albopictus* . Insect Biochem. Mol. Biol. 122, 103386. 10.1016/j.ibmb.2020.103386 32315743

[B41] Noh M. Y. Koo B. Kramer K. J. Muthukrishnan S. Arakane Y. (2016a). *Arylalkylamine N-Acetyltransferase 1* Gene (*TcAANAT1*) Is Required for Cuticle Morphology and Pigmentation of the Adult Red Flour Beetle, *Tribolium castaneum* . Insect Biochem. Mol. Biol. 79, 119–129. 10.1016/j.ibmb.2016.10.013 27816487

[B42] Noh M. Y. Muthukrishnan S. Kramer K. J. Arakane Y. (2016b). Cuticle Formation and Pigmentation in Beetles. Curr. Opin. Insect Sci. 17, 1–9. 10.1016/j.cois.2016.05.004 27720067

[B43] Paulo D. F. Williamson M. E. Arp A. P. Li F. Sagel A. Skoda S. R. (2019). Specific Gene Disruption in the Major Livestock Pests *Cochliomyia hominivorax* and *Lucilia cuprina* Using CRISPR/Cas9. G3 (Bethesda) 9 (9), 3045–3055. 10.1534/g3.119.400544 31340950 PMC6723136

[B44] Rezende G. L. Martins A. J. Gentile C. Farnesi L. C. Pelajo-Machado M. Peixoto A. A. (2008). Embryonic Desiccation Resistance in *Aedes aegypti*: Presumptive Role of the Chitinized Serosal Cuticle. BMC Dev. Biol. 8, 82. 10.1186/1471-213X-8-82 18789161 PMC2561029

[B45] Riedel F. Vorkel D. Eaton S. (2011). Megalin-dependent Yellow Endocytosis Restricts Melanization in the *Drosophila* cuticle. Development 138 (1), 149–158. 10.1242/dev.056309 21138977

[B46] Schmidt C. A. Comeau G. Monaghan A. J. Williamson D. J. Ernst K. C. (2018). Effects of Desiccation Stress on Adult Female Longevity in *Aedes aegypt*i and *Ae. albopictus* (Diptera: Culicidae): Results of a Systematic Review and Pooled Survival Analysis. Parasites Vectors 11 (1), 267. 10.1186/s13071-018-2808-6 29695282 PMC5918765

[B47] Shirai Y. Ohde T. Daimon T. (2021). Functional Conservation and Diversification of *Yellow-y* in Lepidopteran Insects. Insect Biochem. Mol. Biol. 128, 103515. 10.1016/j.ibmb.2020.103515 33387638

[B48] Shirataki H. Futahashi R. Fujiwara H. (2010). Species-specific Coordinated Gene Expression and Trans-regulation of Larval Color Pattern in Three Swallowtail Butterflies. Evol. Dev. 12 (3), 305–314. 10.1111/j.1525-142X.2010.00416.x 20565541

[B49] Sugumaran M. (2002). Comparative Biochemistry of Eumelanogenesis and the Protective Roles of Phenoloxidase and Melanin in Insects. Pigment Cell Res 15 (1), 2–9. 10.1034/j.1600-0749.2002.00056.x 11837452

[B50] Sukhralia S. Verma M. Gopirajan S. Dhanaraj P. S. Lal R. Mehla N. (2019). From Dengue to Zika: the Wide Spread of Mosquito-Borne Arboviruses. Eur. J. Clin. Microbiol. Infect. Dis. 38 (1), 3–14. 10.1007/s10096-018-3375-7 30267170

[B51] Valencia M. D. P. Miller L. H. Mazur P. (1996). Permeability of Intact and Dechorionated Eggs of the *Anopheles* Mosquito to Water Vapor and Liquid Water: A Comparison with *Drosophila* . Cryobiology 33 (1), 142–148. 10.1006/cryo.1996.0014 8812093

[B52] Wang Y. Huang Y. Xu X. Liu Z. Li J. Zhan X. (2020). CRISPR/Cas9‐based Functional Analysis of Yellow Gene in the Diamondback Moth, *Plutella xylostella* . Insect Sci. 28, 1504–1509. 10.1111/1744-7917.12870 32893952 PMC8518405

[B53] Weaver S. C. Reisen W. K. (2010). Present and Future Arboviral Threats. Antiviral Res. 85 (2), 328–345. 10.1016/j.antiviral.2009.10.008 19857523 PMC2815176

[B54] Wittkopp P. J. True J. R. Carroll S. B. (2002). Reciprocal Functions of the *Drosophila* Yellow and Ebony Proteins in the Development and Evolution of Pigment Patterns. Development 129 (8), 1849–1858. 10.1242/dev.129.8.1849 11934851

[B55] Wu X. Zhan X. Gan M. Zhang D. Zhang M. Zheng X. (2013). Laccase2 Is Required for Sclerotization and Pigmentation of *Aedes albopictus* Eggshell. Parasitol. Res. 112 (5), 1929–1934. 10.1007/s00436-013-3349-8 23455937

