# Supplementary material for: Yellow-y Functions in Egg Melanization and Chorion Morphology of the Asian Tiger Mosquito, Aedes albopictus
Source: Front Cell Dev Biol. 2021 Dec 16;9:769788. doi: 10.3389/fcell.2021.769788 (PMC8716798; doi:10.3389/fcell.2021.769788)
Supplement: Supplementary file 1 [file DataSheet1.pdf]

Table S1. Accession numbers of insect Yellow-y used for the amino acid sequence alignment.

Order	Name	Species	Accession number
Diptera	**AalY-y**	*Aedes albopictus*	MN702767
	**AaeY-y**	*Aedes aegypti*	EAT41553
	**AgaY-y**	*Anopheles gambiae*	XP_316854
	**DmY-y**	*Drosophila melanogaster*	NP_476792
	**MdY-y**	*Musca domestica*	ASK51659
	**BtY-y1**	*Bactrocera tryoni*	XP_039962702
	**BdY-y1**	*Bactrocera dorsalis*	QEL52204
	**BlY-y1**	*Bactrocera latifrons*	XP_018802479
	**BtY-y2**	*Bactrocera tryoni*	XP_039962703
	**BdY-y2**	*Bactrocera dorsalis*	XP_011214781
	**BlY-y2**	*Bactrocera latifrons*	XP_018802480
Lepidoptera	**PxY-y**	*Papilio xuthus*	NP_001298746
	**BmY-y**	*Bombyx mori*	BAH11146
	**SlY-y**	*Spodoptera litura*	XP_022829407
	**SfY-y**	*Spodoptera frugiperda*	XP_035431942
	**PxyY-y**	*Plutella xylostella*	CAD7473413
	**AiY-y**	*Agrotis ipsilon*	Supplementary data in Chen et al., 2018
Coleoptera	**TcY-y**	*Tribolium castaneum*	GU111770
	**TmY-y**	*Tenebrio molitor*	QGQ59668
	**LdY-y**	*Leptinotarsa decemlineata*	ATB56361
Hymenoptera	**NvY-y**	*Nasonia vitripennis*	NP_001154977
	**AmY-y**	*Apis mellifera*	NP_001091693
Hemiptera	**OfY-y**	*Oncopeltus fasciatus*	AMW91813
	**PbY-y**	*Platymeris biguttatu*	QEO33343

Table S2. Percentage of amino acid sequence identity/(similarity) of insect Yellow-y proteins.

	AalY-y	AaeY-y	AgaY-y	DmY-y	MdY-y	BtY-y1	BdY-y1	BlY-y1	BtY-y2	BdY-y2	BlY-y2	PxY-y	BmY-y	SlY-y	SfY-y	PxyY-y	AiY-y	TcY-y	TmY-y	LdY-y	NvY-y	AmY-y	OfY-y	PbY-y
**AalY-y**		94.9	79.8	58.0	62.5	60.6	60.6	60.3	67.7	67.8	68.6	57.9	58.4	51.7	61.2	53.4	50.3	64.9	60.3	63.2	62.0	60.7	50.6	52.0
**AaeY-y**	(98.2)		78.6	59.5	64.4	63.6	63.6	63.7	68.1	63.9	69.1	58.0	54.8	51.8	51.8	56.1	50.5	64.4	60.5	61.9	62.1	61.0	51.3	52.1
**AgaY-y**	(88.6)	(88.8)		57.7	60.1	62.5	62.6	61.9	69.2	64.1	70.1	57.4	55.1	56.4	54.3	51.3	50.8	64.5	61.2	63.8	63.6	60.1	56.5	50.9
**DmY-y**	(78.0)	(80.6)	(80.2)		73.1	72.8	73.1	73.3	69.8	68.8	69.4	58.2	62.0	50.3	49.9	50.1	48.8	59.6	59.5	59.3	60.8	61.0	50.5	50.2
**MdY-y**	(83.1)	(84.3)	(81.5)	(86.3)		71.1	71.1	70.1	71.0	68.8	71.5	54.0	60.7	52.6	52.6	52.6	49.7	63.8	61.9	61.3	61.7	63.4	50.7	52.9
**BtY-y1**	(79.1)	(82.5)	(84.1)	(84.2)	(83.3)		98.0	96.7	77.4	76.1	75.8	56.8	55.3	52.4	52.6	52.4	51.6	62.0	62.1	61.9	62.7	62.3	55.6	51.2
**BdY-y1**	(78.9)	(82.5)	(84.1)	(84.2)	(83.3)	(99.6)		96.6	77.9	76.7	75.8	57.4	55.5	52.4	52.6	52.9	51.6	61.0	60.8	61.1	62.4	61.8	55.1	51.0
**BlY-y1**	(80.7)	(82.7)	(84.0)	(84.2)	(83.0)	(98.7)	(98.7)		75.9	75.2	75.4	57.6	56.6	52.4	52.2	52.4	51.4	62.2	61.4	61.8	62.7	61.1	55.1	51.7
**BtY-y2**	(91.1)	(91.4)	(91.6)	(83.7)	(86.1)	(85.5)	(85.5)	(84.8)		93.7	93.3	53.0	63.1	52.0	59.1	54.7	59.4	60.2	59.5	57.3	60.3	61.5	52.0	50.4
**BdY-y2**	(91.7)	(86.8)	(88.6)	(83.9)	(85.4)	(86.1)	(86.3)	(85.7)	(98.4)		92.7	51.9	61.9	55.1	51.9	54.9	59.1	61.2	59.3	56.6	60.1	60.9	52.0	49.4
**BlY-y2**	(91.5)	(92.0)	(92.3)	(83.9)	(85.0)	(85.7)	(85.9)	(85.4)	(98.2)	(99.0)		58.3	62.9	52.0	52.0	58.5	56.9	61.0	60.3	58.6	60.1	60.4	51.7	51.0
**PxY-y**	(79.2)	(80.0)	(80.0)	(80.5)	(75.8)	(80.4)	(80.4)	(80.6)	(76.7)	(77.2)	(83.9)		69.2	63.9	64.1	61.9	71.9	58.9	60.1	59.8	59.9	58.2	50.1	51.5
**BmY-y**	(79.8)	(76.7)	(78.7)	(82.7)	(80.3)	(79.3)	(79.5)	(79.6)	(88.1)	(88.1)	(87.2)	(84.2)		68.5	68.8	62.9	64.6	61.0	61.4	60.8	59.9	61.0	51.8	52.6
**SlY-y**	(76.2)	(76.5)	(81.3)	(74.4)	(76.3)	(76.3)	(76.7)	(76.7)	(78.5)	(82.1)	(79.2)	(81.3)	(88.3)		95.6	62.3	80.2	59.6	60.5	58.2	60.0	56.9	49.8	53.0
**SfY-y**	(84.3)	(76.8)	(78.8)	(74.0)	(76.0)	(76.7)	(77.1)	(77.4)	(85.5)	(79.4)	(79.2)	(82.8)	(88.7)	(99.8)		62.4	78.1	60.1	61.0	59.2	60.5	56.6	48.6	52.0
**PxyY-y**	(76.2)	(76.5)	(75.1)	(74.4)	(77.2)	(78.0)	(78.0)	(78.0)	(82.4)	(82.8)	(87.1)	(81.7)	(81.1)	(81.4)	(82.3)		62.0	61.2	58.8	59.3	58.2	56.5	50.1	52.6
**AiY-y**	(74.3)	(75.4)	(74.5)	(73.5)	(74.5)	(75.7)	(76.5)	(75.9)	(85.7)	(85.7)	(83.9)	(91.1)	(86.2)	(90.2)	(90.0)	(83.5)		59.4	61.0	59.8	60.6	56.9	53.5	52.7
**TcY-y**	(84.9)	(84.5)	(85.9)	(83.8)	(86.3)	(85.7)	(85.5)	(85.5)	(84.9)	(86.0)	(85.2)	(82.0)	(84.0)	(82.5)	(82.8)	(85.6)	(82.4)		74.6	66.1	60.4	59.7	56.9	57.9
**TmY-y**	(83.4)	(84.0)	(84.9)	(84.4)	(86.8)	(87.9)	(87.9)	(87.3)	(86.7)	(87.4)	(86.7)	(82.3)	(85.4)	(83.4)	(83.9)	(84.8)	(83.6)	(91.1)		67.7	61.7	60.8	53.4	55.4
**LdY-y**	(82.8)	(82.6)	(85.2)	(84.4)	(85.6)	(87.0)	(86.8)	(86.6)	(83.6)	(84.4)	(86.4)	(82.9)	(84.7)	(81.9)	(82.4)	(85.9)	(83.1)	(86.5)	(87.8)		61.5	58.5	54.5	54.6
**NvY-y**	(84.8)	(84.7)	(86.9)	(86.7)	(85.9)	(86.4)	(86.4)	(86.4)	(86.2)	(86.7)	(86.2)	(82.4)	(83.2)	(83.3)	(83.6)	(81.6)	(83.7)	(83.9)	(82.9)	(80.8)		75.6	52.6	54.4
**AmY-y**	(84.8)	(85.1)	(84.6)	(84.1)	(84.1)	(84.6)	(84.9)	(83.5)	(84.4)	(84.4)	(83.6)	(82.8)	(83.2)	(81.7)	(81.6)	(81.5)	(82.8)	(80.5)	(81.0)	(80.4)	(91.2)		52.2	53.1
**OfY-y**	(76.0)	(75.8)	(80.9)	(75.7)	(76.7)	(81.6)	(81.6)	(81.3)	(79.0)	(78.8)	(79.1)	(73.1)	(75.9)	(74.8)	(73.7)	(73.8)	(78.3)	(82.1)	(80.7)	(79.5)	(78.2)	(78.5)		62.6
**PbY-y**	(81.8)	(82.1)	(80.8)	(79.2)	(80.5)	(80.5)	(80.2)	(80.7)	(81.1)	(81.1)	(81.5)	(78.9)	(78.6)	(77.1)	(76.4)	(78.8)	(77.1)	(78.9)	(81.0)	(81.1)	(80.3)	(80.2)	(85.9)	

```
   1  atgtggaagtctgtggtctgcgtggcgttgatcggtgccttcggggccgtcagcggaacg   60
   1   M  W  K  S  V  V  C  V  A  L  I  G  A  F  G  A  V  S  G  T    20
  61  tccaagctgcaggaacgctacagctggcgccagttggactttgtgttcccgaaccagcag  120
  21   S  K  L  Q  E  R  Y  S  W  R  Q  L  D  F  V  F  P  N  Q  Q    40
 121  ctcaaacagcaggccctggccagtggggactacgtgccgaccaacggactcccggtcgga  180
  81   L  K  Q  Q  A  L  A  S  G  D  Y  V  P  T  N  G  L  P  V  G   100
 181  atcgaacggtgggagaacaagctctttgtgtccgtgccaagatggaaggatggtatccca  240
 101   I  E  R  W  E  N  K  L  F  V  S  V  P  R  W  K  D  G  I  P   120
 241  tcaaccctgaactacatcgacatgaatcagacgccgtcgggatcgcccccgttgatcccg  300
 121   S  T  L  N  Y  I  D  M  N  Q  T  P  S  G  S  P  P  L  I  P   140
 301  tatccaagctgggccaacaacgtggctggagactgtcagaacggtctgtcgaccgtgtac  360
 141   Y  P  S  W  A  N  N  V  A  G  D  C  Q  N  G  L  S  T  V  Y   160
 361  cgtatcaaggctgacaagtgcggacgcctctgggttctggacaccggtaccgttggaatc  420
 161   R  I  K  A  D  K  C  G  R  L  W  V  L  D  T  G  T  V  G  I   180
 421  ggaaacaccacccagcagctgtgcccgtacgcgctgaacatctttgacctcaagaccaac  480
 181   G  N  T  T  Q  Q  L  C  P  Y  A  L  N  I  F  D  L  K  T  N   200
 481  acccgtctgcgacgatacgaactgcgtgctgaagacaccaaccagaacaccttcatcgcc  540
 201   T  R  L  R  R  Y  E  L  R  A  E  D  T  N  Q  N  T  F  I  A   220
 541  aacattgccatcgacatgggacgcagctgcgacgacacctttgcctacatgtccgacgaa  600
 221   N  I  A  I  D  M  G  R  S  C  D  D  T  F  A  Y  M  S  D  E   240
 601  ttgggctacggattgattgcctactcgtttgagaagaacaagtcctggcggttcgagcac  660
 241   L  G  Y  G  L  I  A  Y  S  F  E  K  N  K  S  W  R  F  E  H   260
 661  agcttcttcttcccggatcctctgcgtggagacttcaacgtcgccggcctgaacttccaa  720
 261   S  F  F  F  P  D  P  L  R  G  D  F  N  V  A  G  L  N  F  Q   280
 721  tggggtgaggaaggtatcttcggaatgtcgctgtccccaatgcaagccgacggtttccgt  780
 281   W  G  E  E  G  I  F  G  M  S  L  S  P  M  Q  A  D  G  F  R   300
 781  accatgtacttctcaccactggctagccaccgtgaattcatggtctccactcaggtcctc  840
 301   T  M  Y  F  S  P  L  A  S  H  R  E  F  M  V  S  T  Q  V  L   320
 841  cgcgatgaagaaggcgctgaagagagcttccacaagttcacctacctgaaggaacgagga  900
 321   R  D  E  E  G  A  E  E  S  F  H  K  F  T  Y  L  K  E  R  G   340
 901  cccaacagccacactacatcccgagttatgagcgagatcggactgcagctgttcaacctg  960
 341   P  N  S  H  T  T  S  R  V  M  S  E  I  G  L  Q  L  F  N  L   360
 961  atcgaccagaacgccgtcggatgctggcattcgtcgctgccttacagccccgagaaccac 1020
 361   I  D  Q  N  A  V  G  C  W  H  S  S  L  P  Y  S  P  E  N  H   380
1021  ggaatcgtcgaccgcgacgatgtcgaactggtcttccctgctgacgtcaagatcgatttc 1080
 381   G  I  V  D  R  D  D  V  E  L  V  F  P  A  D  V  K  I  D  F   400
1081  gaagaaaacgtgtgggtcatttccgaccgtatgccggtgttcctcatcgccgagctggac 1140
 401   E  E  N  V  W  V  I  S  D  R  M  P  V  F  L  I  A  E  L  D   420
1141  tacagcgatgttaacttccgcatcttcaccgctcctctgagcaccctggtcgcaggaacc 1200
 421   Y  S  D  V  N  F  R  I  F  T  A  P  L  S  T  L  V  A  G  T   440
1201  gtctgcgatgtcgccccatcgctccgaccaggtgccatccagtccaaattcggtggatcc 1260
 441   V  C  D  V  A  P  S  L  R  P  G  A  I  Q  S  K  F  G  G  S   460
1261  gatctgacaacctacccaggaaccaccctactcccagccgggtacagccagcccatcagc 1320
 461   D  L  T  T  Y  P  G  T  T  L  L  P  A  G  Y  S  Q  P  I  S   480
1321  tacaccccgacctcctacgccccgactgttgcccccgtgaccaagtacacatccgccccg 1380
 481   Y  T  P  T  S  Y  A  P  T  V  A  P  V  T  K  Y  T  S  A  P   500
1381  gcagcgtacgatcaccccacatcccacatgtacacgacccaggaatatccaacgaccgcc 1440
 501   A  A  Y  D  H  P  T  S  H  M  Y  T  T  Q  E  Y  P  T  T  A   520
1441  aaggcgtaccacttcaacaagtaccacaacgtggagtaccaatcgcacggaaacggccaa 1500
 521   K  A  Y  H  F  N  K  Y  H  N  V  E  Y  Q  S  H  G  N  G  Q   540
1501  gccgactaccacttcggccacggaggacattaccacggtggtcatgaccatggcggccat 1560
 541   A  D  Y  H  F  G  H  G  G  H  Y  H  G  G  H  D  H  G  G  H   560
1561  gactacggccacgatcacggatactacggcggcggagagcaccgccggtactggggagct 1620
 561   D  Y  G  H  D  H  G  Y  Y  G  G  G  E  H  R  R  Y  W  G  A   580
1621  ggccccaagaagcctgaagcctggaagcagcagctgtactag                   1662
 581   G  P  K  K  P  E  A  W  K  Q  Q  L  Y  -                     593
```

Fig. S1. Nucleotide and deduced amino acid sequences of AalY-y. Predicted secretion signal peptide sequences and the putative major royal jelly protein domains (MRJP) are underlined and boxed, respectively. Predicted *N*-glycosylation sites are highlighted in gray.

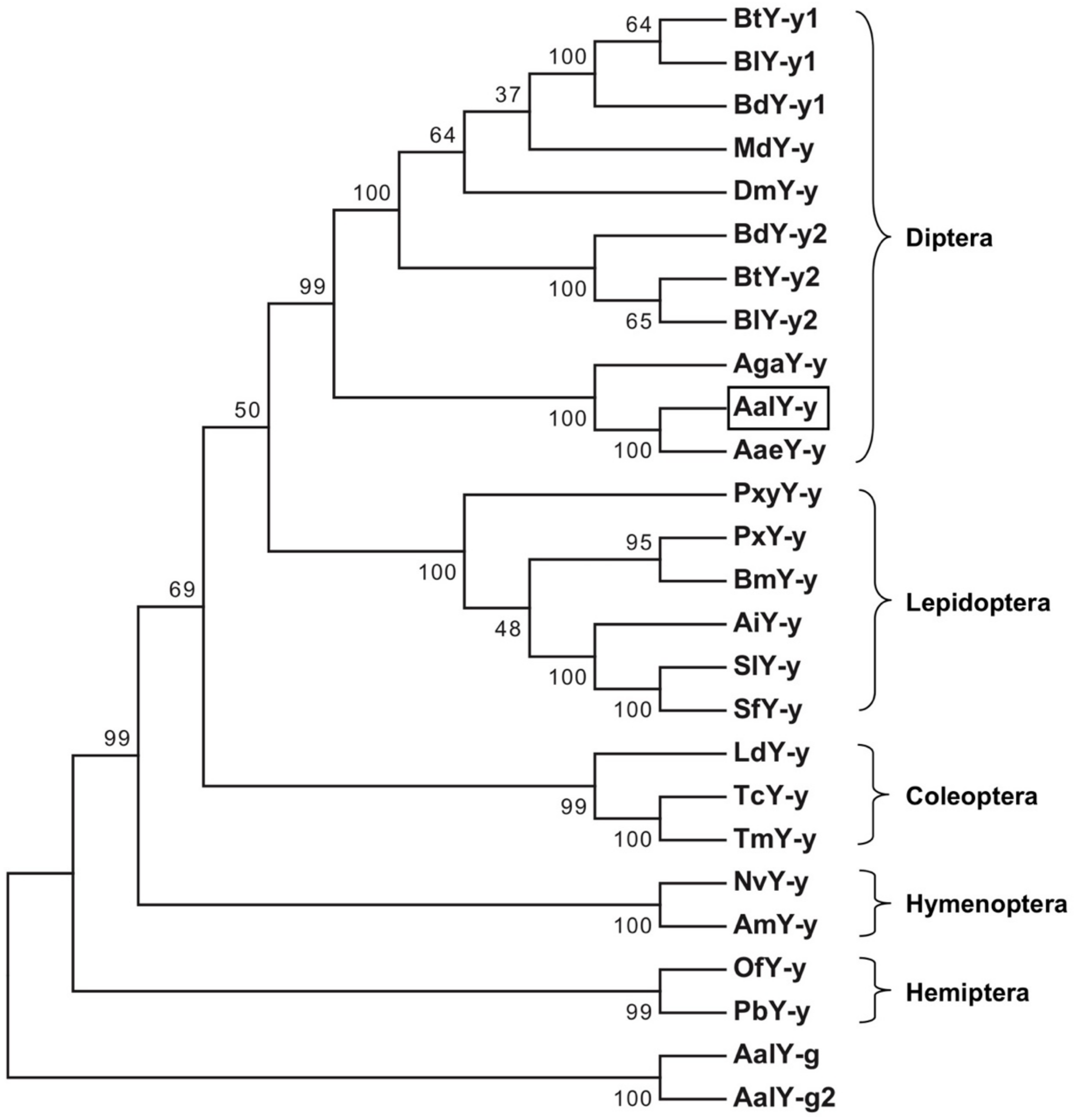

Fig. S2. Phylogenetic analysis of insect Yellow-y proteins. The phylogenetic tree was constructed by MEGA7 software using the Unweighted Pair Group Method with Arithmetic Mean (UPGMA). Numbers by each branch indicate results of bootstrap analysis of 1000 replications. AalY-g and AalY-g2 (accession number: MH970513 and MH936662, respectively) from *Ae. albopcitus* are used for outgroup. See Table S1 for the accession numbers and abbreviations of Yellow-y proteins used.

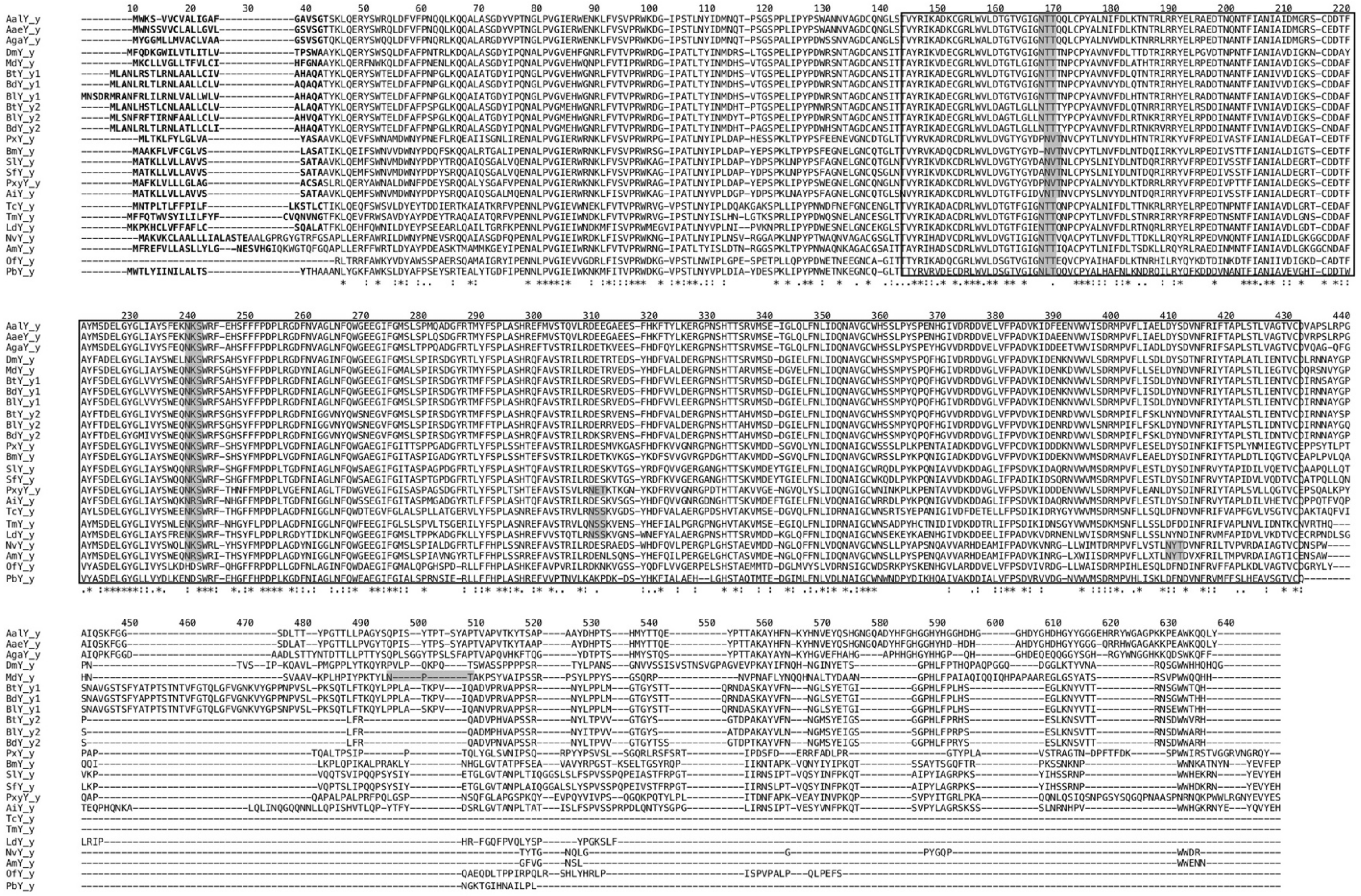

Fig. S3. Amino acid sequence alignment of insect Yellow-y proteins. Multiple sequence alignment of insect Yellow-y proteins was made using ClustalW software. Predicted signal peptides and putative MRJP domain are bolded and boxed, respectively. Putative *N*-glycosylation sites are shaded. Symbols below the aligned amino acid sequences indicate identical (*), highly conserved (:), and conserved residues (.), respectively. See Table S1 for the accession numbers and abbreviations of Yellow-y proteins used.
